# Achieving sustainable development goals with primary care physicians

**DOI:** 10.51866/ed0008

**Published:** 2024-01-01

**Authors:** Ping Foo Wong, Wai Khew Lee, V Paranthaman

**Affiliations:** 1 MBBS, Dr Fam Med, MAFP, FRACGP, Klinik Kesihatan Cheras Baru, Jalan 16, Kampung Cheras Baru, Off Jalan Kuari, Cheras, Kuala Lumpur, Malaysia. Email: dr.wongpingfoo@moh.gov.my; 2 MBBS, M. Fam Med., MSc., Klinik Kesihatan Luyang, Sabah, Malaysia.; 3 MBBS, MMed, Klinik Kesihatan Greentown, Jalan Raja Musa Aziz, Ipoh, Perak, Malaysia.

## Introduction

The commentary by Prof. Renganathan on the Sustainable Development Goals (SDGs)^1^ highlights Malaysia’s performance on Goal 3 and underscores the role of and implications for primary care physicians. This article further details the practices by public primary care physicians in Malaysia.

The SDGs were adopted by the United Nations in 2015. They comprise of 17 goals with 169 targets that are inter-linked and inter-connected. The SDGs aim to end poverty and protect the planet as well as ensure that all people live in peace, good health, justice and prosperity. Goal 3 is directly related to health — to ensure healthy lives and promote well-being for all ages — with 13 targets and 28 indicators.^2^ The Ministry of Health Malaysia has shown steadfast commitment in attempting to achieve the global goals for the country by 2030.

All four areas identified by Prof. Renganathan where primary care physicians can contribute towards achieving the SDGs (i.e. clinical, community, practice management and advocacy) are not new but are worth re-emphasising. As primary care physicians see patients across the spectrum, the nine outcome targets identified are within the work scope, and all primary care physicians have the responsibility to help work towards achieving them.

Herein, we highlight an important target for Malaysia — reducing premature mortality from non-communicable diseases (NCDs). NCDs are the leading cause of death globally and locally. They are one of the major challenges hindering the country from achieving the goal. Target 3.4 aims to reduce premature mortality from NCDs by one-third through prevention and treatment and to promote mental health and well-being. In line with this target, the World Health Organization (WHO) Global Action Plan for the Prevention and Control of Non-Communicable Diseases 2013–2020 was developed and implemented. At the local level, the National Strategic Plan for Non-Communicable Diseases 2016–2025 was produced. Table 1 summarise the progress of indicators and targets for non-communicable diseases in Malaysia.

**Table 1:** Progress of indicators and targets for non-communicable diseases.^3-6^

No.	Indicator	Global target	Malaysia		
			Baseline	Current	Target (2025)
1.	Risk of premature mortality from cardiovascular diseases, cancer, diabetes or chronic respiratory diseases	25% relative reduction	20%	18.5% (2019)	15%
2.	Prevalence of current tobacco use in people aged ≥15 years	30% relative reduction	23%	21.3% (2019)	15%
3.	Mean population intake of sodium	30% relative reduction	8.7 g	7.9 g (2019)	6.0 g
4.	Prevalence of insufficient physical activity	10% relative reduction	35.2%	25.1% (2019)	30.0%
5.	Harmful use of alcohol (prevalence of heavy episodic drinking)	10% relative reduction	≤1.2%	10% (2019)	≤1.2%
6.	Prevalence of raised blood pressure	25% relative reduction	32.3%	30% (2019)	26.0%
7.	Prevalence of diabetes and obesity	Halt the rise	≤15%	18.3^*^ (2019)	≤15%

* diabetes

## Current practices

### 1. Clinical care

The provision of clinical care to people living with NCDs has been the core service in primary care settings under the Ministry of Health Malaysia (MOH). The management of NCDs and chronic diseases forms the bulk of daily burden in health clinics. A report by the WHO showed that diabetes accounted for approximately 45% of Malaysia’s RM 9.65 billion direct healthcare costs from three chronic conditions (diabetes, cardiovascular diseases and cancer) in 2017.^7^ Clinical care is delivered beyond geographical and economical boundaries. Primary care physicians and their teams work together to ensure that people living with NCDs achieve optimum targets and control of their diseases. The provision of clinical care involves screening to detect complications early. An example is using fundus cameras in primary care clinics to screen for diabetic retinopathy.

### 2. Expanded-scope programme

Many expanded-scope programmes are implemented to further strengthen efforts in addressing the risk factors of NCDs: smoking cessation programme, alcohol use screening and intervention programme, weight loss programme and enhanced primary healthcare programme. Each programme comes with specific activities and modules. In addition, cancer screening programmes such as Pap smear/human papilloma virus screening for cervical cancer, immunochemical faecal occult blood test for colorectal cancer and breast examination and mammography for breast cancer are offered to all clients who visit government health clinics. Although mammography may not be available in health clinics, clear clinical pathways have been drawn out to ensure patients can obtain such services in nearby hospitals. Mental health screening is also conducted using the WHOQOL checklist.

### 3. Clinical audit

The National Diabetes Registry was an initiative taken by the Ministry of Health Malaysia in 2009, with the aim of monitoring the quality of care provided to people with diabetes managed at primary care clinics. Initially, data were collected manually, but a web-based data collection system was then used in 2011. In recent years, audits on hypertension and asthma have been introduced and initiated. These audits are mainly conducted by primary care physicians and are important in monitoring the standards of care and the clinical outcomes.

### 4. Community screening

Primary care clinics are located near communities. Primary care teams in health clinics work closely with communities to promote a healthy lifestyle. One community activity implemented is Komuniti Sihat Pembina Negara (KOSPEN), wherein community health volunteers are trained to become health enablers who introduce and facilitate healthy living practices among their communities. Primary care physicians also work closely with the Clinic Health Advisory Panel, government agencies and non-governmental organisations to carry out health screening in communities regularly. The National Health Screening Initiative, an ambitious programme initiated by the Ministry of Health Malaysia that aims to screen for NCDs among individuals aged above 40 years, is conducted in health clinics and communities. Free health screening for the underprivileged via the PeKa B40 programme, which is subsidised by the government, is implemented by primary care physicians both in government and private sectors.

### 5. Health education and advocacy

Primary care physicians take care of the health of families in communities. Good rapport and relationship between primary care physicians and communities enable health promotion through health education and advocacy. Primary care physicians frequently deliver health talks and become advocates of health through various health camps and media outlets.

### 6. Capacity training and leadership role

A strong primary care team ensures a good care outcome for people living with NCDs. Primary care physicians provide training and equip their primary care teams with updated knowledge and skills in managing people living with NCDs. They assume the role of a leader in primary care clinics, advancing unit organisation.

## Way forward

Primary care physicians play an important role in realising the SDGs for the country. However, their efforts can be strengthened with optimum resources, including manpower, medications, facilities and budget allocations. Objective lifestyle assessment and coaching can be further improved on top of pharmacological interventions for managing NCDs.

## References

[1] Renganathan E, Davies P (2023). Sustainable Development Goals and the role of and implications for primary care physicians.. Malays Fam Physician..

[2] World Health Organization. (2013). Global Action Plan for the Prevention and Control of Noncommunicable Diseases 2013-2020..

[3] Ministry of Health. (2016). National Strategic Plan for Non-Communicable Disease (NSPNCD) 2016-2025..

[4] Institute for Public Health, National Institutes of Health, Ministry of Health Malaysia. (2020). National Health and Morbidity Survey (NHMS) 2019: Vol. I: NCDs - Non-Communicable Diseases: Risk Factors and Other Health Problems..

[5] World Health Organization. (2022). Noncommunicable Diseases Progress Monitor 2022..

[6] Disease Control Division Non-Communicable Disease Section, Department of Public Health. (2013). National Diabetes Registry Report, Volume 1: 2009-2012..

[7] Ministry of Health Malaysia. (2022). Direct HealthCare Cost of Noncommunicable Diseases in Malaysia..

